# Variation in leisure sport conflicts and coping strategies depending on participation type and proximity during the COVID-19 pandemic

**DOI:** 10.3389/fpubh.2023.1093541

**Published:** 2023-02-27

**Authors:** Young-Jae Kim, Kyu-lee Shin, Seung-Woo Kang

**Affiliations:** ^1^Department of Physical Education, Chung-Ang University, Seoul, Republic of Korea; ^2^Department of Sports Science, Seoul National University of Science and Technology, Seoul, Republic of Korea

**Keywords:** coping strategy, COVID-19, leisure sports activities, leisure conflicts, spatial proximity

## Abstract

**Introduction:**

New conflict types have arisen in leisure sports activities due to social regulations designed to address COVID-19. We analyze the differences in conflict-inducing factors and coping strategies across various types of leisure sports and levels of spatial proximity.

**Methods:**

Korean adults aged between 20 and 60 years, who had participated in leisure sports activities since the COVID-19 outbreak in January 2020, were surveyed, and 508 responses were collected for analysis. The differences in leisure sports conflicts and coping strategies across the types of leisure sports participation and spatial proximity were tested.

**Results:**

The results show that conflict due to prejudice was higher in typical indoor sports activities, such as Pilates, yoga, and gym workouts, whereas conflict due to competition or not observing etiquette was higher in indoor golf. Second, conflict due to prior expectations and prejudice was high in outdoor sports activities, such as jogging and hiking. Finally, all participants showed avoidance behavior, but it was observed more frequently in outdoor sports than indoor sports.

**Discussion:**

The study reveals how much leisure conflict is induced by various types of leisure sports participation, particularly during outdoor activities, which usually feature a relatively low density of participants. It underscores the necessity of developing structural approaches to resolving leisure conflicts in dangerous spaces or requiring intensive management and creating new leisure sports activities.

## 1. Introduction

The Korean government restricted both indoor and outdoor activities and gatherings during the COVID-19 pandemic (1). These restrictions led people to engage in leisure sports activities, such as at-home and individual workouts, differently than they did in the past. Full participation in leisure sports activities decreased with the continued constraints on gatherings and indoor activities (2–5).

Scholars have argued that a strong immune system is necessary to prevent being infected with COVID-19. Sports and outdoor activities with relatively few constraints are important for maintaining and fortifying immunity. Baker and Simpson (6) and Simpson and Katsanis (7) emphasize the need to maintain optimum immune functioning through outdoor leisure sports, which have lower infection risks compared to indoor sports because infection spreads *via* respiratory droplets. Consequently, outdoor leisure sports activities became essential during this period due to the constraints on indoor activities and individuals' anxiety over becoming infected.

Participation in leisure sports activities, which had been increasing in the 5 years prior to COVID-19, declined after the outbreak due to fears of infection, restrictions on the use of space, and the curtailment of leisure activities as a preventive measure (8–10). Accordingly, participation decreased in indoor leisure sports, such as gym workouts, yoga, Pilates, and swimming (11), and increased in outdoor leisure events, such as walking, hiking, and soccer (12).

As evidenced by previous outbreaks, decision-making is linked to ethical issues when health is at risk (13). Ethical issues arise when values and norms are conflicted or when they no longer apply. Conflicts also occur in sporting situations among individuals or between other groups and may include disagreements about the rules or structures guiding individuals' professional activities (14). Action cannot be avoided in these circumstances. Therefore, decisions must be made (15), and making difficult decisions in challenging circumstances can lead to moral distress (16). Drawing from these studies, ethical conflict due to prejudice can be defined as “one or more negative self-directed emotions or attitudes that arise in response to perceived participation in a situation that is perceived as morally undesirable” (17).

However, as participation in outdoor leisure activities increased, the activity areas became more crowded and conflict situations increased (18). With multitudes being packed into limited spaces for outdoor sports, it became difficult for people to maintain appropriate social distance, and many felt uncomfortable using the facilities when they were crowded. Failure to maintain physical distance increases anxiety about infection transmission; hence, crowding of the activity space prevented participants from having a pleasant experience. Choon (19) argues that the perception of crowding in leisure sports activity spaces is affected more by the surrounding environment than the actual crowding of the space itself.

Most modern people have undergone extreme restraints on outdoor activities due to the risk of infectious diseases, their psychological responses should be studied with a focus on wellbeing to establish a relationship between perfectionism, resilient resources, and the psychological wellbeing of athletes who experience different confinement situations. However, those who have not undergone restraints have possibly had their sports routines changed due to country-specific restrictions, such as the closure of facilities and difficulty in accessing sports materials (20).

In general, barriers to physical activity are often related to time constraints and limiting social factors, for example, some students have had difficulty finding time to engage in physical activity while managing homework and other obligations. That said, competitive environments have been identified as creating potential conflict among peers. Likewise, individuals expressed fear of not knowing how to play sports or anxiety about infectious diseases in a competitive environment (21).

Conflicts arising out of the rapid increase in outdoor leisure sports participants in limited spaces have been primarily related to etiquette, space management, and space planning (22, 23). For example, not wearing a mask correctly, talking too much, or drinking beverages during exercise increase others' anxiety about exposure to the virus and cause conflicts among participants (24, 25).

With the risk of infection somewhat reduced, people seemed to be going out more than ever, and congestion has been reported at overcrowded recreational areas, hiking trailheads, and popular tourist destinations. Similar interests in various media and SNS have brought people together, and based on this, observations and problems have been identified among those participating in such activities; however, the overall aim is to solve the rapidly growing interest in outdoor activities while also creating a relationship between recreational activists. This reveals several concerns and challenges, including how to manage ecological pressures and social tensions. Issues with current developments such as overcrowding, new visitors, problematic behavior, social distancing, event cancellations, and conflicts between different groups of users, including visitors and landowners, must be identified. Derks et al. (26) specifically discuss day-to-day management challenges and opportunities. These demands increase and diversify and can develop into conflict if not managed carefully; therefore, it may be advantageous to manage them intensively and observe and study the behavior of visitors during periods when rushing into nature is most prevalent (27).

Nevertheless, the conflicts vary with participation type (outdoor vs. indoor) and spatial proximity. Before the pandemic, most conflicts involved the inconvenience of using leisure sports facilities. However, since the onset of the pandemic, new factors, such as the discomfort or displeasure caused by participants using the facilities, have been found to disrupt leisure sports participation. In sum, excessive spatial proximity in leisure sports activities may be harmful (28).

Although lockdown restrictions vary among countries and have not been enforced simultaneously, some regulations have been mandatory worldwide (home confinement, closure of cultural and social events, remote work, online education, movement restrictions, social distancing, and physical activity restrictions) (29). The concept of multidimensional infectious disease vulnerability is more relevant due to increased threats to personal health and disrupted stress responses; therefore, anxiety regarding sports spaces and concerns regarding the risk of infection both imply a state of vulnerability (30, 31). During 2020, outdoor spaces saw evident changes with members seeking outdoor gym facilities, yoga classes migrating from studios to parks and forests, as well as new clubs and *ad hoc* walking and running groups being formed. Furthermore, commuting and other everyday chores were undertaken for recreation and leisure and seen as opportunities for exercise (32, 33). By analyzing and better understanding how these changes were perceived, a new perspective in leisure and social science theories can emerge to explore in advance the various conflict situations that appear when participating in leisure activities (34, 35).

Jacob and Schreyer (36) identify that conflict in a crowded space is influenced by activity type, the importance of specific activity resources, concentration on the environment or activity, and users' tolerance of different lifestyles. They further point out that conflict factors or the extent thereof may vary between indoor and outdoor activities depending on the crowdedness of the space. Therefore, an increase in spatial proximity, or the reduction of physical distance among participants, also aggravates the degree of conflict or stress felt by the participants, and their responses to conflicts may vary depending on these circumstances.

Therefore, our research objective in this paper is to test the contention that the degree of conflict varies depending on the spatial proximity that occurs in indoor and outdoor settings; to test this, four factors of existing conflict were used together: (1) Conflict due to prejudice; (2) Conflict due to competition; (3) Prior expectations; and (4) Conflict due to not observing etiquette.

In other words, we emphasize spatial proximity as an important new factor in conflict. Because of the nature of the COVID-19 pandemic, we posit a research hypothesis that the “space” in sports activities will be a major factor predicting conflict, because people fear becoming infected and their perception of risk increases as their distance from other participants narrows. The spatial proximity concept we use is derived from Edward Hall's work (37).

Regarding spatial proximity, Hall (37) classifies the interpersonal distances among humans (the relative distances between people) into four zones based on social interactions. The first is an “intimate distance” (within 120 cm), common among family members or lovers, which involves significant risk of direct infection *via* droplets. A “personal distance” (120–350 cm) is maintained by friends or colleagues, and one may be exposed to infection when many people occupy space at this distance from one another. A “social distance” (3.5–7.5 m) is larger but allows people to hear each other when communicating by voice; this distance was recommended during the pandemic. Finally, when people communicate across a “public distance” (over 7.5 m), they must use a loud voice.

Many studies have explored conflicts due to the social distancing measures implemented during the pandemic (38–40). Moreover, responses to conflicts due to COVID-19 have varied depending on individual traits (41–44). Therefore, the conflicts and responses among participants in indoor and outdoor sports activities may vary depending on their spatial proximity.

Additionally, participants' satisfaction levels with leisure activities differ depending on conflict coping strategies. The ultimate goal of leisure is to improve individual happiness and quality of life (45). However, an individual's subjective happiness derived from leisure activities is an aggregate of positive and negative experiences. Therefore, achieving subjective happiness through leisure requires increasing the positive experiences and decreasing the negative experiences from such activities (46). This requires us to identify the conflict factors among the participants which will help reduce their consequent negative experiences.

Considering the emergence of new conflict types from the social regulations associated with COVID-19, we investigate the specific inconveniences and negative emotions associated with conflicts in leisure sports activities. We analyze how conflict patterns and respondents' coping methods vary according to the type of leisure sports participation (indoor vs. outdoor) and spatial proximity. By examining conflicts that arise due to proximity, this study can offer new perspectives on the operation and management of leisure sports environments.

## 2. Materials and methods

### 2.1. Data

We conducted a survey using a convenience sample of adults aged between 20 and 60 years, who had frequently participated in leisure sports activities after the COVID-19 outbreak in January 2020. The survey was conducted online from May 12–18, 2021, using the Korean research company Macromill EMBRAIN. Before the survey, potential participants were provided with information regarding the research's content, purpose, and ethical considerations, and informed consent was secured from those willing to participate. We gave respondents a mobile coupon worth 3,000 won (2–3 dollar) as an incentive for participating in this study. A total of 550 individuals participated, indicating a response rate of 92.31%. After 42 responses were excluded due to duplicate responses or omissions, a sample of 508 responses was used for the analysis. The survey was approved by the (blinded for review - Chung-Ang University Research Ethics Committee) Institutional Review Board (IRB) (No. 1041078-202010-HRSB-313-01).

### 2.2. Participants

Supplementary Table A1 in the appendices reports the respondents' demographic characteristics. Out of 508 respondents, 236 (46.5%) were male, 272 (53.5%) were female, and the average age was 39.72 years. Regarding average monthly income, 195 participants (38.4%) earned between KRW 2.01–4 million, 110 participants (21.7%) earned between KRW 4.01–6 million, 72 participants (14.2%) earned KRW 1 million or less, 74 participants (14.5%) earned at least KRW 6.01 million, and 57 participants (11.2%) earned KRW 1.01–2 million. Each respondent's leisure sports participation was classified as indoor and outdoor activities. Among indoor activities, most of the respondents engaged in swimming (44; 20.0%) followed by indoor golf (43; 19.5%) and ball sports (futsal, volleyball, basketball, etc.) (43; 19.5%). Among outdoor activities, most of the respondents participated in badminton (80; 27.8%) followed by ball sports (soccer, baseball, basketball, and so on) (72; 25.0%).

### 2.3. Measurement instrument

The questionnaire used in this study included items on sports participation types, leisure conflicts and coping strategies, and spatial proximity in the COVID-19 context (21, 31). Demographic characteristics included sex, age, marital status, and income. SPSS 26.0 was used to analyze the data. After creating the first draft of the questionnaire, the content validity was assessed by a professor of leisure studies and two PhD researchers in the same field to check the suitability of the items. Thereafter, the validity and reliability of the constructs were analyzed. One-way analysis of variance (ANOVA) was conducted to assess the differences in leisure sports conflicts and coping strategies between types of participation and levels of spatial proximity. This was done as an ANOVA (F-test) was employed by Ho (46), complemented with a *post hoc* Scheffe's test (if the variances were found to be homogeneous according to a Levene's test) or a *post hoc* Games–Howell test (if the variances were not homogeneous). The level of significance adopted was p < 0.05.

### 2.4. Leisure conflict

The leisure conflict measurement used in this study is based on Vaske et al. (47). It was used by Schroeder et al. (48) to investigate conflict characteristics for different leisure situations and is comprised of four types of conflicts: those due to prior expectations, competition, prejudice, and not observing etiquette. Due to prior expectations, the first conflict type includes three items describing people's expectations before engaging in leisure sports activities. The second type, conflicts due to competition, includes four items measuring conflicts caused by the participants. The third type, conflicts due to prejudice, includes two items on prejudice against other participants. The final type, conflicts due to not observing etiquette, includes five items. The Kaiser-Meyer-Olkin (KMO) test was conducted to verify the distribution of the data and its suitability for factor analysis; the KMO value was 0.888, indicating adequate sampling. The chi-square approximation for Bartlett's test of sphericity was 5,949.643 (*p* < 0.001). Varimax rotation (orthogonal rotation) was used, and the total cumulative variance was 76.695 (see Supplementary Table A2).

### 2.5. Coping strategy

Measurement instruments for coping strategies for conflicts have been based on the theories of goal interference and crowding (36, 49). In this study, the coping strategy scale used by Schneider et al. (50) was revised and extended to include three items, each on avoidance behavior and resolution behavior, rated on a five-point Likert scale. The standard KMO test was used to verify the adequacy of the data for factor analysis and normal distribution, revealing a measure of 0.706. The chi-square approximation for Bartlett's test of sphericity was 834.240 (*p* < 0.001). Varimax rotation (orthogonal rotation) was used, and the total cumulative variance was 62.692 (Supplementary Table A3).

### 2.6. Spatial proximity

Spatial proximity was measured based on Hall (37), using the classification standard used by Kim and Kang (51) for distances required for leisure sports activities. The four categories were intimate distance (within 1.2 m), personal distance (1.2–3.5 m), social distance (3.5–7.0 m), and public distance (7.5 m and above). The sports activities in which the distance between the instructor and the participants or among the participants is close, such as Pilates, yoga, and personal training, were coded as having an intimate distance of 1.2 m. For activities such as swimming and gym workouts, the required distance varied depending on participant density within the space; generally, the required distance was about 2 m, which falls within the personal distance interval of 1.2–3.5 m. Activities, such as badminton or tennis that involved courts required at least a 2 m distance between courts; thus, the social distance of 3.5–4.7 m was maintained. Meanwhile, jogging, hiking, and golf were conducted without limits in an outdoor space; thus, the public distance of 7.5 m and above was maintained.

## 3. Results

The results of the analysis validating the differences in leisure conflicts and coping strategies according to the type of participation and spatial proximity in the COVID-19 context are presented below. The two types of participation are indoor and outdoor.

### 3.1. Differences in leisure conflicts by type of participation

Table 1 reports the results of the analysis of differences in conflict-inducing factors for indoor leisure sports participation. Indoor golf (2.44 ± 0.75) and swimming (2.43 ± 0.71) showed higher levels of conflict due to competition than Pilates and yoga (1.83 ± 0.73) (F = 2.932, *p* = 0.05). Meanwhile, gym workout (3.05 ± 0.98) showed higher levels of conflict due to prejudice than indoor badminton (2.15 ± 1.08) and ball sports (2.36 ± 0.98) (F = 3.868, df = 5, *p* = 0.001). Pilates and yoga (3.00 ± 1.07) also showed higher levels of conflict due to prejudice than indoor badminton (2.15 ± 1.08). Conflicts due to not observing etiquette were greater in indoor golf (2.17 ± 0.66) than in Pilates and yoga (1.53 ± 0.55) (F = 3.275, df = 5, *p* = 0.001). The mean difference in conflict due to prior expectations for indoor leisure sports participation was not statistically significant.

**Table 1:** Differences in leisure conflicts among indoor leisure sports (*N* = 220).

**Variable**	**N (%)**	**Conflict due to prior expectations**	**F/t**	***Post hoc* test**	**Conflict due to competition**	**F/t**	***Post hoc* test**
**Indoor leisure sports activities**		**M** ±**SD**			**M** ±**SD**		
Indoor badminton^a^	26 (11.8)	3.64 ± 0.61	1.773		2.29 ± 0.81	2.932^*^	2 > 5
Indoor golf^b^	43 (19.5)	3.89 ± 0.54			2.44 ± 0.75		
Swimming^c^	44 (20.0)	3.54 ± 0.67			2.43 ± 0.71		
Gym workout^d^	28 (12.7)	3.55 ± 0.70			2.20 ± 0.78		
Pilates-yoga^e^	36 (16.4)	3.64 ± 0.60			1.83 ± 0.73		
Ball sports^f^	43 (19.5)	3.69 ± 0.59			2.23 ± 1.01		
Total	220						
**Variable**	**N (%)**	**Conflict due to prejudice**	**F/t**	***Post hoc*** **test**	**Conflict due to not observing etiquette**	**F/t**	***Post hoc*** **test**
**Indoor leisure sports activities**		**M** ±**SD**			**M** ±**SD**		
Indoor badminton^a^	26 (11.8)	2.15 ± 1.08	3.868^***^	4 > 1, 6; 5 > 1	1.83 ± 0.86	3.275^***^	2 > 5
Indoor golf^b^	43 (19.5)	2.57 ± 0.90			2.17 ± 0.66		
Swimming^c^	44 (20.0)	2.73 ± 0.96			2.00 ± 0.63		
Gym workout^d^	28 (12.7)	3.05 ± 0.98			1.75 ± 0.78		
Pilates-yoga^e^	36 (16.4)	3.00 ± 1.07			1.53 ± 0.55		
Ball sports^f^	43 (19.5)	2.36 ± 0.98			1.92 ± 0.98		
Total	220						

^***^p < 0.001, ^*^p < 0.05. M, mean; SD, standard deviation.

Table 2 presents the results of the analysis of differences in conflict-inducing factors for outdoor leisure sports participation. Jogging (4.16 ± 0.58) showed higher levels of conflict due to prior expectations than badminton (3.88 ± 0.51), while hiking (4.30 ± 0.66) showed higher levels of conflict due to prior expectations than both badminton (3.88 ± 0.51) and ball sports (3.96 ± 0.48) (F = 4.688, df = 4, *p* = 0.001). Conflicts due to prejudice were greater in jogging (3.25 ± 0.89) than in badminton (2.44 ± 0.83), ball sports (2.27 ± 1.01), and golf (2.12 ± 0.81) (F = 13.029, df = 4, *p* = 0.001). Finally, the mean difference in conflicts due to not observing etiquette for outdoor leisure sports participation was not statistically significant.

**Table 2:** Differences in leisure conflicts among outdoor leisure sports (*N* = 288).

**Variable**	**N (%)**	**Conflict due to prior expectations**	**F/t**	***Post hoc* test**	**Conflict due to competition**	**F/t**	***Post hoc* test**
**Outdoor leisure sports activities**		**M** ±**SD**			**M** ±**SD**		
Badminton^a^	80 (27.8)	3.88 ± 0.51	4.688^***^	c > a; d>a, e	2.28 ± 0.84	0.887	
Golf^b^	56 (19.4)	4.06 ± 0.43			2.17 ± 0.83		
Jogging^c^	53 (18.4)	4.16 ± 0.58			2.05 ± 0.83		
Hiking^d^	27 (9.4)	4.30 ± 0.66			2.01 ± 0.79		
Ball sports^e^	72 (25.0)	3.96 ± 0.48			2.16 ± 0.81		
Total	288						
**Variable**	**N (%)**	**Conflict due to prejudice**	**F/t**	***Post hoc*** **test**	**Conflict due to not observing etiquette**	**F/t**	***Post hoc*** **test**
**Outdoor leisure sports activities**		**M** ±**SD**			**M** ±**SD**		
Badminton^a^	80 (27.8)	2.44 ± 0.83	13.029^***^	c > a, b, e	1.93 ± 0.64	0.653	
Golf^b^	56 (19.4)	2.12 ± 0.81			1.79 ± 0.77		
Jogging^c^	53 (18.4)	3.25 ± 0.89			1.78 ± 0.72		
Hiking^d^	27 (9.4)	2.67 ± 1.00			1.95 ± 0.75		
Ball sports^e^	72 (25.0)	2.27 ± 1.01			1.80 ± 0.80		
Total	288						

^***^p < 0.001. M, mean; SD, standard deviation.

### 3.2. Differences in leisure conflicts depending on spatial proximity by type of leisure sports

Table 3 presents the results of the analysis of differences in conflict-inducing factors due to spatial proximity in indoor leisure sports participation. Personal distance (2.24 ± 0.75) and social distance (2.40 ± 0.85) showed higher levels of conflict due to competition than public distance (1.48 ± 0.58) (F = 4.569, df = 3, *p* = 0.01). However, the mean differences in conflicts due to prior expectations, prejudice, and not observing etiquette for indoor leisure sports participation were not statistically significant.

**Table 3:** Differences in leisure conflicts depending on spatial proximity among indoor leisure sports (*N* = 220).

**Variable**	**N (%)**	**Conflict due to prior expectations**	**F/t**	***Post hoc* test**	**Conflict due to competition**	**F/t**	***Post hoc* test**
**Proximity in indoor leisure sports activities**		**M** ±**SD**			**M** ±**SD**		
Intimate distance^α^	17 (7.7)	3.82 ± 0.75	0.483		2.07 ± 1.04	4.569^***^	β, γ > δ
Personal distance ^β^	111 (50.5)	3.65 ± 0.61			2.24 ± 0.75		
Social distance ^γ^	81 (36.8)	3.67 ± 0.62			2.40 ± 0.85		
Public distance ^δ^	11 (5.0)	3.58 ± 0.62			1.48 ± 0.58		
Total	220						
**Variable**	**N (%)**	**Conflict due to prejudice**	**F/t**	***Post hoc*** **test**	**Conflict due to not observing etiquette**	**F/t**	***Post hoc*** **test**
**Indoor leisure sports participation proximity**		**M** ±**SD**			**M** ±**SD**		
Intimate distance^α^	17 (7.7)	2.26 ± 1.16	1.619		2.00 ± 1.08	1.996	
Personal distance ^β^	111 (50.5)	2.57 ± 0.94			1.87 ± 0.71		
Social distance ^γ^	81 (36.8)	2.78 ± 1.03			1.96 ± 0.78		
Public distance ^δ^	11 (5.0)	2.86 ± 1.38			1.38 ± 0.67		
Total	220						

^***^p < 0.001. M, mean; SD, standard deviation.

Table 4 presents the results of the analysis of differences in conflict-inducing factors due to spatial proximity in outdoor leisure sports participation. Public distance (2.08 ± 0.87) showed higher levels of conflict due to competition than personal distance (2.28 ± 0.81) and social distance (2.17 ± 0.82) (F = 3.755, df = 3, *p* = 0.01). Conflict due to prejudice was greater in the personal distance (2.45 ± 0.88), social distance (2.55 ± 0.97), and public distance (2.93 ± 1.03) categories than intimate distance (1.79 ± 1.09) (F = 6.653, df = 3, *p* = 0.001). Personal distance (1.90 ± 0.67) showed higher levels of conflict due to not observing etiquette than intimate distance (1.42 ± 0.81) (F = 2.706, df = 3, *p* = 0.05). Finally, the mean difference in conflict due to prior expectations for outdoor leisure sports participation was not statistically significant.

**Table 4:** Differences in leisure conflicts depending on spatial proximity among outdoor leisure sports (*N* = 288).

**Variable**	**N (%)**	**Conflict due to prior expectations**	**F/t**	***Post hoc* test**	**Conflict due to competition**	**F/t**	***Post hoc* test**
**Outdoor leisure sports participation proximity**		**M** ±**SD**			**M** ±**SD**		
Intimate distance^α^	21 (7.3)	3.98 ± 0.62	0.312		1.64 ± 0.74	3.755^**^	β, γ > α
Personal distance^β^	118 (41.0)	4.03 ± 0.52			2.28 ± 0.81		
Social distance^γ^	113 (39.2)	4.01 ± 0.50			2.17 ± 0.82		
Public distance^δ^	36 (12.5)	4.10 ± 0.63			2.08 ± 0.87		
Total	288						
**Variable**	**N (%)**	**Conflict due to prejudice**	**F/t**	***Post hoc*** **test**	**Conflict due to not observing etiquette**	**F/t**	***Post hoc*** **test**
**Proximity in outdoor leisure sports activities**		**M** ±**SD**			**M** ±**SD**		
Intimate distance^α^	21 (7.3)	1.79 ± 1.09	6.653^***^	β, γ, δ > α	1.42 ± 0.81	2.706^*^	β > α
Personal distance ^β^	118 (41.0)	2.45 ± 0.88			1.90 ± 0.67		
Social distance ^γ^	113 (39.2)	2.55 ± 0.97			1.85 ± 0.76		
Public distance ^δ^	36 (12.5)	2.93 ± 1.03			1.88 ± 0.72		
Total	288						

^***^p < 0.001, ^**^p < 0.01, ^*^p < 0.05. M, mean; SD, standard deviation.

### 3.3. Verifying the difference in coping strategies depending on type of leisure sports participation

Table 5 presents the analysis of the differences in coping strategies for indoor leisure sports participation. Avoidance behavior was the highest in indoor golf (3.57 ± 0.62) and the lowest in indoor badminton (3.23 ± 0.82) but was not statistically significant. Similarly, resolution behavior was the highest in indoor golf (3.88 ± 0.47) and the lowest in indoor badminton (3.67 ± 0.67) but was not statistically significant.

**Table 5:** Differences in coping strategies among indoor leisure sports (*N* = 220).

**Variable**	**N (%)**	**Avoidance behavior**	**F/t**	***Post hoc* test**	**Resolution behavior**	**F/t**	***Post hoc* test**
**Indoor leisure sports participation**		**M** ±**SD**			**M** ±**SD**		
Indoor badminton^a^	26 (11.8)	3.23 ± 0.82	1.235		3.67 ± 0.67	0.796	
Indoor golf^b^	43 (19.5)	3.57 ± 0.62			3.88 ± 0.47		
Swimming^c^	44 (20.0)	3.53 ± 0.71			3.76 ± 0.69		
Gym workout^d^	28 (12.7)	3.50 ± 0.84			3.87 ± 0.58		
Pilates·yoga^e^	36 (16.4)	3.45 ± 0.87			3.85 ± 0.85		
Ball sports^f^	43 (19.5)	3.27 ± 0.71			3.69 ± 0.64		
Total	220						

M, mean; SD, standard deviation.

Table 6 presents the analysis of the differences in coping strategies for outdoor leisure sports participation. Badminton (3.69 ± 0.62) showed higher avoidance behavior than ball sports (3.33 ± 0.89) (F = 2.743, df = 4, *p* = 0.05). Resolution behavior was the highest in ball sports (3.96 ± 0.56) and the lowest in badminton (3.73 ± 0.64), but the difference was not statistically significant.

**Table 6:** Difference in coping strategies for outdoor leisure sports participation (*N* = 288).

**Variable**	**N (%)**	**Avoidance behavior**	**F/t**	***Post hoc* test**	**Resolution behavior**	**F/t**	***Post hoc* test**
**Outdoor leisure sports participation**		**M** ±**SD**			**M** ±**SD**		
Badminton^a^	80 (27.8)	3.69 ± 0.62	2.743^*^	a> e	3.73 ± 0.64	1.709	
Golf^b^	56 (19.4)	3.48 ± 0.78			3.85 ± 0.48		
Jogging^c^	53 (18.4)	3.66 ± 0.81			3.79 ± 0.65		
Hiking^d^	27 (9.4)	3.68 ± 0.76			3.88 ± 0.47		
Ball sports^e^	72 (25.0)	3.33 ± 0.89			3.96 ± 0.56		
Total	288						

^***^p < 0.001, ^*^p < 0.05. M, mean; SD, standard deviation.

### 3.4. Verifying the difference in coping strategies depending on spatial proximity in leisure sports participation

Table 7 presents the analysis of differences in coping strategies depending on spatial proximity in indoor leisure sports participation. Social distance (3.65 ± 0.65) showed higher levels of avoidance behavior than intimate distance (2.95 ± 1.00) and personal distance (3.34 ± 0.73) (F = 6.848, df = 3, *p* = 0.001). The mean difference in resolution behavior due to spatial proximity in leisure sports participation was not statistically significant.

**Table 7:** Differences in coping strategies depending on spatial proximity among indoor leisure sports (*N* = 220).

**Variable**	**N (%)**	**Avoidance behavior**	**F/t**	***Post hoc* test**	**Resolution behavior**	**F/t**	***Post hoc* test**
**Proximity in indoor leisure sports participation**		**M** ±**SD**			**M** ±**SD**		
Intimate distance^α^	17 (7.7)	2.95 ± 1.00	23.096^***^	γ>α, β	3.84 ± 0.64	0.085	
Personal distance ^β^	111 (50.5)	3.34 ± 0.73			3.81 ± 0.53		
Social distance ^γ^	81 (36.8)	3.65 ± 0.65			3.82 ± 0.61		
Public distance ^δ^	11 (5.0)	3.97 ± 0.93			3.84 ± 0.66		
Total	220						

^***^p < 0.001. M, mean; SD, standard deviation.

Table 8 reports the results of the analysis of differences in coping strategies depending on spatial proximity in outdoor leisure sports participation. Social distance (3.64 ± 0.66) showed higher levels of avoidance behavior than intimate distance (3.05 ± 1.11). Public distance (4.09 ± 0.76) showed higher levels of avoidance behavior than intimate distance (3.05 ± 1.11), personal distance (3.39 ± 0.72), and social distance (3.64 ± 0.66) (F = 12.149, df = 3, *p* = 0.001). The mean difference in resolution behavior due to spatial proximity in leisure sports activities was not statistically significant.

**Table 8:** Differences in coping strategies depending on spatial proximity among outdoor leisure sports (*N* = 288).

**Variable**	**N (%)**	**Avoidance behavior**	**F/t**	***Post hoc* test**	**Resolution behavior**	**F/t**	***Post hoc* test**
**Proximity in outdoor leisure sports participation**		**M** ±**SD**			**M** ±**SD**		
Intimate distance^α^	21 (7.3)	3.05 ± 1.11	12.149^***^	Γ > α; δ > α, β, γ	3.71 ± 0.69	0.879	
Personal distance^β^	118 (41.0)	3.39 ± 0.72			3.80 ± 0.53		
Social distance^γ^	113 (39.2)	3.64 ± 0.66			3.86 ± 0.61		
Public distance^δ^	36 (12.5)	4.09 ± 0.76			3.94 ± 0.60		
Total	288						

^***^p < 0.001. M, mean; SD, standard deviation.

## 4. Discussion

This study examined differences in leisure conflicts and coping strategies between indoor and outdoor sports. It sought to examine the causes of conflicts in leisure sports activities and provide resolutions by exploring the two sports activity types that generate conflicts among the participants and analyzing their coping strategies. The research aimed to help increase healthy and sound participation in leisure sports activities in response to the continuing COVID-19 pandemic.

There were several key findings. First, typical indoor activities such as Pilates, yoga, and gym workouts showed high levels of conflict due to prejudice. Conflicts due to the competition and not observing etiquette were high in indoor golf but lower in outdoor golf. Conflicts due to competition were relatively low since partitions had been installed between sports equipment and people used the equipment at different times (52). Moreover, yoga and Pilates showed relatively low levels of conflict due to competition because these activities were carried out by appointment only, limiting the use of activity space. Indoor golf showed high levels of conflict from competition or conflict due to not observing etiquette, and “the risk of being infected by others” was added as a new conflict factor (53). By contrast, conflicts of this kind were relatively low for outdoor golf because sufficient distance could be maintained between participants in an outdoor space. The result indicates that the type and level of conflict varies depending on the available space, even for the same activity. Furthermore, it indicates that spatial proximity must be included in the analysis of conflicts in leisure sports activities.

Second, outdoor activities such as jogging and hiking showed high conflicts due to prior expectations or prejudice. Conflicts due to prejudice were perhaps caused by many people gathering in outdoor spaces for sports, which was considered safer than indoor sports during the pandemic (54–56). Thus, outdoor spaces became so crowded that any contact between participants could disturb individual activity and perceptions, and a new leisure conflict emerged. Previous studies of leisure conflict mostly argue that usage patterns and spatial characteristics of leisure activity space can have a negative effect on leisure satisfaction (57, 58). However, recent studies of leisure conflict cover various conflicts, such as collisions between joggers and bike riders in Hangang Park (59), whether hikers can share mountain trails with bike or motorcycle riders, and conflicts between people fishing and people water skiing (60). These studies reveal new leisure conflicts that emerge from the growth of the number of participants in outdoor leisure sports.

The analysis of the impact of spatial proximity revealed, contrary to expectations, greater conflict in the personal distance and social distance categories during outdoor activities than during indoor activities. Additionally, a high level of conflict during outdoor activities indicates that merely maintaining personal or social distance was insufficient despite the extra space afforded by the outdoor environment. This suggests that participants' satisfaction with sports or activities decreases when they cannot maintain unencumbered space around themselves, one of the benefits they anticipate from an outdoor space. According to Kim and Kang (51) participants enjoying leisure sports feel it is crowded when they are closer to one another, increasing their perceived risk of infection; hence, they deliberately chose outdoor leisure sports or activity locations that were relatively free from crowd and risk.

Conflicts also emerged when the greater distance was maintained between participants, as in the case of public distance. This points to other conflict factors besides spatial proximity to the National Medical Center in outdoor leisure sports or activities, even if there is sufficient physical distance. These factors include crowding that occurs when the facility accommodates more people than it can, difficulties in using the facility, and concerns about infection due to failure to observe facility-related etiquette. Conflicts in outdoor activities emerge when users do not observe safety etiquette and prioritize only their health (61–63).

The study also analyzed the differences in coping strategies of participants across types of leisure sports activities and levels of spatial proximity. While the participants of both indoor and outdoor sports or activities showed avoidance behavior (39, 64, 65), it was more frequently found in outdoor sports, suggesting that people feel a sense of difference from participants engaging in activities different from them, and conflicts arise in the process of each struggling to blend in with the other. Thus, the participants took actions such as avoiding other participants, moving to a different place, trying to maintain a specific distance from others, or adjusting their schedules to avoid crowding (66–68). Meanwhile, the results regarding resolution behavior were insignificant (69). The participants passively approached the stress and confusion they faced in new leisure conflicts (70, 71).

The results indicate an urgent need to devise solutions to emerging conflicts in indoor and outdoor leisure sports so that participants can enjoy more positive and beneficial experiences. This study explored different levels of conflict for each type of participation and the coping strategies that leisure sports participants used in situations requiring social distancing. Despite the expectation that the relatively low population density in outdoor spaces would lead to lower conflict, contrary results were found. Therefore, future studies must specifically examine the causes of conflicts separately for indoor and outdoor leisure sports and activities. Moreover, frameworks should be established to resolve conflicts that arise in spaces that are dangerous or require intensive management, thereby creating new environments that increase participation in leisure or sports activities. It is also necessary to lay the groundwork for more research in this domain.

## 5. Conclusions

The COVID-19 pandemic has decreased participation in indoor leisure sports and activities and increased outdoor ones. However, the changes have resulted in various conflicts, such as anxiety about contracting infections during leisure sports activities, particularly considering the rise in participation. This study examined the differences in conflicts based on the type of leisure sports participation (indoor and outdoor) and spatial proximity, assessing the differences in participants' coping strategies. The major findings are as follows.

First, compared to indoor activities, the participants sensed fewer conflicts in outdoor activities during which they could maintain social distancing. Second, the conflict-inducing factors varied depending on whether the activity took place indoors or outdoors; however, as participants experienced more crowding in outdoor activities, conflicts arose. Finally, people participating in leisure sports activities chose avoidance when coping with leisure conflicts, instead of taking active measures, regardless of whether the activities took place indoors or outdoors.

Based on these results, it is necessary to consider specific plans, other than avoidance, to resolve leisure conflicts in both indoor and outdoor activities. Standards must be established for activity spaces that allow participants to enjoy positive and beneficial experiences. The literature on leisure sports and activities has focused only on the positive effect these activities have on people's lives. However, post-COVID-19, research must consider the negative aspects of such activities, such as increased stress and the spread of infection. This study has established new directions for developing research on leisure sports conflicts to provide the groundwork for increasing people's participation in leisure sports and activities in the COVID-19 context.

## Data availability statement

The original contributions presented in the study are included in the article/Supplementary material, further inquiries can be directed to the corresponding author.

## Ethics statement

The studies involving human participants were reviewed and approved by the Chung-Ang University Ethics Committee reviewed and approved the protocol of this study. The patients/participants provided their written informed consent to participate in this study.

## Author contributions

Material preparation, data collection, and analysis were performed by S-WK and Y-JK. The first draft of the manuscript was written by S-WK. All authors commented on subsequent versions of the manuscript, read and approved the final manuscript, and contributed to the study's conception and design.
