# Supplementary material for: Variation in leisure sport conflicts and coping strategies depending on participation type and proximity during the COVID-19 pandemic
Source: Front Public Health. 2023 Feb 27;11:1093541. doi: 10.3389/fpubh.2023.1093541 (PMC10008941; doi:10.3389/fpubh.2023.1093541)
Supplement: Supplementary file 1 [file Table_1.docx]

# Appendices

**Table A1**

*Participant Characteristics*

| Variable | | | N (%) |
| --- | --- | --- | --- |
| Sex | | Male | 236(46.5) |
|  |  | Female | 272(53.5) |
|  |  | Total | 508 |
| Age | | 20s | 108(21.3) |
|  |  | 30s | 121(23.8) |
|  |  | 40s | 136(26.8) |
|  |  | 50s | 143(28.1) |
|  |  | Total | 508 |
| Marital status | | Single | 196(38.6) |
|  |  | Married | 297(58.4) |
|  |  | Other | 15(3.0) |
|  |  | Total | 508 |
| Average monthly income | | KRW 1 million or less | 72(14.2) |
|  |  | KRW 1.01–2 million | 57(11.2) |
|  |  | KRW 2.01–4 million | 195(38.4) |
|  |  | KRW 4.01–6 million | 110(21.7) |
|  |  | Over KRW 6 million | 74(14.5) |
|  |  | Total | 508 |
| Type of leisure sports participation | Indoor leisure sports | Indoor badminton | 26(11.8) |
|  |  | Indoor golf driving range | 43(19.5) |
|  |  | Swimming | 44(20.0) |
|  |  | Gym workout | 28(12.7) |
|  |  | Pilates, yoga | 36(16.4) |
|  |  | Ball sports (futsal, volleyball, basketball, etc.) | 43(19.5) |
|  |  | Total | 220 |
|  | Outdoor leisure sports | Badminton | 80(27.8) |
|  |  | Golf | 56(19.4) |
|  |  | Jogging | 53(18.4) |
|  |  | Hiking | 27(9.4) |
|  |  | Ball sports (soccer, baseball, basketball, etc.) | 72(25.0) |
|  |  | Total | 288 |
| Spatial proximity | Indoor spatial proximity | Intimate distance (<1.2 m) | 17(7.7) |
|  |  | Personal distance (1.2–3.5 m) | 111(50.5) |
|  |  | Social distance (3.5–7.5 m) | 81(36.8) |
|  |  | Public distance (>7.5 m) | 11(5.0) |
|  |  | Total | 220 |
|  | Outdoor spatial proximity | Intimate distance (<1.2 m) | 21(7.3) |
|  |  | Personal distance (1.2–3.5 m) | 118(41.0) |
|  |  | Social distance (3.5–7.5 m) | 113(39.2) |
|  |  | Public distance (>7.5 m) | 36(12.5) |
|  |  | Total | 288 |

**Table A2**

*Exploratory factor analysis of leisure conflicts*

| Item | | Factor | | | | h^2^ |
| --- | --- | --- | --- | --- | --- | --- |
|  |  | 1 | 2 | 3 | 4 |  |
| Conflict due to not observing etiquette 4 | Other participants are causing damage to surroundings aside from activity space. | .865 | .304 | -.057 | .117 | .857 |
| Conflict due to not observing etiquette 3 | Other participants damage the environment. | .851 | .252 | -.065 | .069 | .796 |
| Conflict due to not observing etiquette 2 | Other participants are doing leisure activities in areas that are off limits. | .838 | .293 | -.084 | .146 | .817 |
| Conflict due to not observing etiquette 5 | Other participants are disturbing repose by making noises (singing loudly) during the activity. | .807 | .230 | -.066 | .113 | .772 |
| Conflict due to not observing etiquette 1 | Other participants are disrupting the leisure space by carrying out leisure activities around others. | .764 | .310 | -.086 | .191 | .723 |
| Conflict due to competition 1 | Other participants are disturbing my leisure activities. | .331 | .859 | -.079 | .088 | .862 |
| Conflict due to competition 2 | My repose is adversely affected. | .269 | .858 | -.077 | .059 | .818 |
| Conflict due to competition 3 | The leisure activity space is crowded. | .292 | .814 | -.054 | .203 | .792 |
| Conflict due to competition 4 | Other participants do not offer me space for leisure activities. | .366 | .767 | -.032 | .094 | .732 |
| Prior expectations 2 | I think the places where leisure activities take place offer mental relaxation. | -.154 | -.189 | .844 | .015 | .772 |
| Prior expectations 3 | I think leisure activities are for enjoyment. | -.147 | -.177 | .816 | -.005 | .718 |
| Prior expectations 1 | I think I can enjoy nature while carrying out leisure activities. | .074 | .181 | .629 | -.188 | .469 |
| Conflict due to prejudice 2 | It is difficult to blend in with other participants. | .128 | .128 | -.043 | .902 | .811 |
| Conflict due to prejudice 1 | I feel a sense of difference from other participants. | .236 | .149 | -.127 | .847 | .848 |
| Reliability | | .929 | .916 | .606 | .804 |  |
| Eigenvalue | | 3.933 | 3.254 | 1.834 | 1.717 |  |
| Variance (%) | | 28.093 | 23.242 | 13.098 | 12.262 |  |
| Cumulative variance (%) | | 28.093 | 51.334 | 64.432 | 76.695 |  |
| KMO=.888, χ^²^=5949.643, df=91, *p*<.001 | | | | | |  |

**Table A3**

*Exploratory factor analysis of coping strategies*

| Item | | Factor | | h^2^ |
| --- | --- | --- | --- | --- |
|  |  | 1 | 2 |  |
| Avoidance behavior 2 | I choose the space by avoiding other participants. | .861 | .090 | .749 |
| Avoidance behavior 1 | I try to retain a certain distance to avoid disturbing other participants. | .849 | .150 | .743 |
| Avoidance behavior 3 | I participate in leisure activities at times when others do not participate. | .742 | .118 | .565 |
| Resolution behavior 2 | I have sufficient prior knowledge. | .143 | .740 | .568 |
| Resolution behavior 3 | I politely apologize when a conflict arises with other participants. | .003 | .801 | .641 |
| Resolution behavior 1 | Etiquette must be made mandatory for a mature culture of leisure activities. | .188 | .678 | .468 |
| Reliability | | .769 | .604 |  |
| Eigenvalue | | 2.069 | 2.069 |  |
| Variance (%) | | 34.481 | 34.481 |  |
| Cumulative variance (%) | | 28.211 | 62.692 |  |
| KMO=.706, χ^²^=834.240, df=15, *p*<.001 | | | |  |
